# Predictors of effectiveness of early intervention on children with intellectual disability: a retrospective cohort study

**DOI:** 10.1186/1471-2431-14-170

**Published:** 2014-07-02

**Authors:** Der-Chung Lai, Chung-Hsin Chiang, Yuh-Ming Hou, Jiun-Horng Liu, Shu-Fen Yao, How-Ran Guo, Yen-Cheng Tseng

**Affiliations:** 1 Department of Physical Medicine and Rehabilitation, Ditmanson Medical Foundation Chia-Yi Christian Hospital, Chia-Yi, Taiwan; 2 Department of Senior Citizen Service Management, Chia Nan University of Pharmacy and Science, Tainan, Taiwan; 3 Department of Psychology and Center for Mind, Brain and Learning, National Chengchi University, Taipei, Taiwan; 4 Department of Psychiatry, Ditmanson Medical Foundation Chia-Yi Christian Hospital, Chia-Yi, Taiwan; 5 Department of Psychiatry, Chimei Medical Center, Liouying, Tainan, Taiwan; 6 Reporting and Referral Center for Children with Developmental Delay of the Chia-Yi City and County, Chia-Yi, Taiwan; 7 Department of Occupational and Environmental Medicine, National Cheng Kung University Hospital, Tainan, Taiwan; 8 Department of Environmental and Occupational Health, National Cheng Kung University, Tainan, Taiwan; 9 Language Education Center, Chang Jung Christian University, Tainan, Taiwan; 10 Department of Business Administration, Chang Jung Christian University, Tainan, Taiwan

## Abstract

**Background:**

The Taiwanese government has been promoting early intervention to children with intellectual disability for years, but data on its effectiveness are limited.

**Methods:**

We recruited children who were treated for intellectual disability at a teaching hospital and had two IQ tests from 2001 to 2005 and used the difference between the two tests as the indicator of effectiveness.

**Results:**

The participants included 23 boys and 13 girls 56.5 ± 5.9 months of age at the first test and 73.4 ± 4.9 months at the second. The IQ increased from 57.0 ± 8.0 to 65.1 ± 12.3 (*p* < 0.001). Multi-variate regressions showed that a low maternal educational level, male gender, and a younger age at the first test were significant independent predictors of the effectiveness.

**Conclusions:**

Early intervention can improve the IQ of children with intellectual disability, and the earlier the intervention the better. The effectiveness is demonstrable in boys and more prominent in children whose mothers had a low educational level.

## Background

According to the Diagnostic and Statistical Manual of Mental Disorders, Fourth Edition (DSM-IV), the diagnostic criteria for mental retardation (MR) are (1) significantly sub-average intellectual functioning, (2) concurrent deficits or impairments in adaptive functioning, and (3) onset before age 18 years [1]. It is the most prevalent major developmental disability [2], with prevalence rates around 1% to 2.5% in the U.S. [2,3]. MR was synonymous with intellectual disability (ID), and most people use the term ID in recent years because of the issues with stigmatizing [4].

Preventing children with ID from becoming permanently disabled and reducing the degree of their permanent disability are important public health issues [5]. Early intervention is a measure to achieve those goals, which is based on the concept that cerebral plasticity is better in the early years of life [6]. While early diagnosis and intervention is a general principle of disease treatment, according to the Children and Youth Welfare Law [7] and its By-laws [8], early intervention for children with ID in Taiwan has some features: (1) performed before 6 years of age, (2) offered to the child as well as the family members, and (3) conducted through team-working among professionals in medicine, social welfare, and education.

The promulgation of the Children Welfare Law [9] in 1993 is a milestone in the promotion of early intervention in Taiwan, which mandates early intervention services provided by the government. Accordingly, the Ministry of the Interior established local Early Intervention Reporting Referral Center (EIRRC), and the Department of Health selected a hospital in each City and County to establish a Child Development Evaluation Center. With the implementation of the National Health Insurance in 1995, hospitals at all levels began to expand the manpower and facilities for early intervention. Consequently, Taiwan has been constructing the early intervention system step by step [10].

Whereas the Taiwan government has paid a lot of attention and money into early intervention, the effectiveness, data on its effectiveness are limited. There are many ways to evaluate the effectiveness of early intervention on children with ID, and IQ tests are the most frequently applied [11-14]. Early intervention has both long-term and short-term effects [15-17]. In the short term, patients who receive early intervention have larger improvement in the IQ scores. Although most studies showed that the increase in IQ faded off gradually after the termination of intervention, long-term follow-ups found that patients receiving early intervention had smaller proportions of those who need special education placements, repeat their grades at school, and have juvenile delinquency, but a larger proportion of those who graduated from high schools. Environmental risk factors such as low income, low maternal education level, and single-parent family have negative impacts on children cognitive development [18-20]. Children with exposure to environmental risk factors are the most vulnerable and require early intervention [13,20], and some studies showed that early intervention was more effective in the children with low maternal education levels [14,19,21].

To assess the current state of early intervention services in Taiwan, we conducted a study in the Chia-Yi area and tried to address four important issues: (1) how much early intervention can improve the IQ of children with ID, (2) whether it is the earlier the better for early intervention, (3) whether more hours per week of early intervention can lead to better effectiveness, and (4) what the impacts of various environmental factors are on the effectiveness of early intervention.

## Methods

### Participants

We recruited children who were treated at the Child Development Evaluation Center of a teaching hospital in Chia-Yi, Taiwan from January 1, 2001 to December 31, 2005. The inclusion criteria are (1) the child had at least two test scores for Wechsler Preschool and Primary Scale of Intelligence-Revised (WPPSI-R) [22] and (2) the psychiatric diagnosis was MR at the time of the first WPPSI-R test. If there were more than two tests, the two that were furthest apart between 3 and 7 years of age were adopted for analysis.

The exclusion criteria are (1) the two tests were less than 1 year apart, (2) the child had received other intelligence or development tests, such as Bayley Scales of Infant Development, Mullen Scales of Early Learning, and Stanford-Binet Intelligence Scale, before the first WPPSI-R test, (3) the child did not reside in the Chia-Yi area, which covers the Chia-Yi City and the Chia-Yi County, and (4) the child was claimed to be a resident of the Chia-Yi area, but no intervention data could be found in the EIRRC of the area. To ensure the comparability [23], we included only participants with at least two scores for the same test. Because the WPPSI-R is the most frequently used intelligence and development test in the Chia-Yi area for pre-school children, we limited our analyses to this test. On the basis of the concept that assessment is the beginning of intervention [24], the time of the first test is approximately the beginning of early intervention Therefore, the second criterion was applied to exclude cases who had received early intervention before the first WPPSI-R test. The reason for applying the third criterion was that information on intervention could not be easily obtained through the EIRRC of the Chia-Yi area.

### Data collection

Through social workers at the EIRRC of the Chia-Yi area, we collected data on the type of interventions and number of hours of each type of intervention between the two tests; income, education levels, and marital statuses of the parents; and the relationship between the patient and the primary care-giver. We classified interventions into four types: (1) treatments at the hospital, which include physical therapy, occupational therapy, speech therapy, and psychotherapy, (2) kindergarten education, (3) pre-school special education, and (4) itinerant teacher services, which is provided at home or school on an individual basis by teachers through the arrangement of the EIRRC. We divided the total number of hours of intervention by the number of weeks between the two tests to calculate the number of hours per week. Furthermore, we collected data on potential environmental risk factors, including low income, low maternal education level, low father’s education level, single-parent family, and grand parenting. We defined “low income” as having a low or a relatively low income family status certified by the government for the purpose of social welfare. Because junior high school education is mandatory in Taiwan, we defined “low maternal education level” and “low father’s education level” as levels below senior high school graduate. We defined “grand parenting” as having grandparent(s) as the primary caregiver(s).

In addition to the first test score (IQ1) and the second test score (IQ2), we collected data on the gender, the ages when the IQ tests were performed, and the psychiatric diagnosis at the time of the first test, which was made according to the DSM-IV, from the medical records at the hospital.

The WPPSI-R used in Taiwan was the Chinese version on which the norm has been established, and all the tests in our study were administered by certified psychologists. We used the full scale IQ score of WPPSI-R, and the participants had a mean of 100 and a standard deviation (SD) of 15 [22]. According to ICD-10 [25,26], we used IQ 50 and IQ 70 as cutoffs and classified IQ into three levels (IQ < 50, IQ 50 to 69, IQ ≥ 70) [27]. We used the difference between IQ1 and IQ2 (IQ2–IQ1) as the indicator of the effectiveness of the intervention. This study was approved by the Institutional Review Board of the Ditmanson Medical Foundation Chia-Yi Christian Hospital.

### Data analysis

We presented descriptive statistics of the variables as mean ± SD or percentage. We evaluated differences in IQ scores using the paired t-test or Wilcoxon signed ranks test when data were compared in pairs, and applied the Mann–Whitney U test when the differences in IQ scores were evaluated by stratified analyses. To identify the predictors of the effectiveness of early intervention and evaluate their effects, we used linear regressions. We conducted the data analyses using SPSS for Windows Version 15.0 and performed statistical tests at the two-tailed significance level of 0.05.

## Results

Of the children who were treated at the Child Development Evaluation Center of the Hospital in the study period, 65 fit the inclusion criteria. Of the 65 children, 5 received the two tests less than 1 year apart, 13 had received other intelligence or development tests before the first WPPSI-R test, 8 were not residents of the Chia-Yi area, and 3 were residents of the Chia-Yi area who had no intervention data. Therefore, the final study population consisted of 36 children, including 23 boys and 13 girls. The mean age was 56.5 ± 5.9 (ranging from 44 to 67) months at the first test, and 73.4 ± 4.9 (ranging from 61 to 83) months at the second test. The gap between the two tests ranged from 12 to 27 months, with an average of 16.9 ± 4.4 months and a median of 16 months.

Most participants received kindergarten education (34 participants, 94.4%) and treatments at the hospital (33, 91.7%), while only 3 (8.3%) each received itinerant teacher services and pre-school special education. Many participants received more than one type of treatment, and the most common mode was both kindergarten education and treatments at the hospital (31, 86.1%). The average hours per week were 24.1 ± 11.0 for kindergarten education and 0.8 ± 0.6 for treatments at the hospital. Among the nine participants at the lowest IQ level, seven (77.8%) received kindergarten education and two (22.2%) received pre-school special education.

As to environmental risk factors, there were 14 participants (38.9%) with a low maternal educational level, 10 (27.8%) with a low father’s educational level, 4 (11.1%) from low income families, 5 (13.9%) from single-parent families, and 6 (16.7%) receiving grand parenting.

The average IQ2 (65.1 ± 12.3, ranging from 42 to 96) was higher than the average IQ1 (57.0 ± 8.0, ranging from 43 to 69) (*p* < 0.001), indicating a significant increase. The increase (IQ2–IQ1) ranged from −11 to 27, with an average of 8.1 ± 9.8. When we classified the IQ scores into three levels, we found 13 (48.1%) of the 27 participants with an IQ1 at the second level improved to one level higher, and 6 of the 9 (66.7%) with an IQ1 at the first level improved to one level higher.

**Figure 1:**
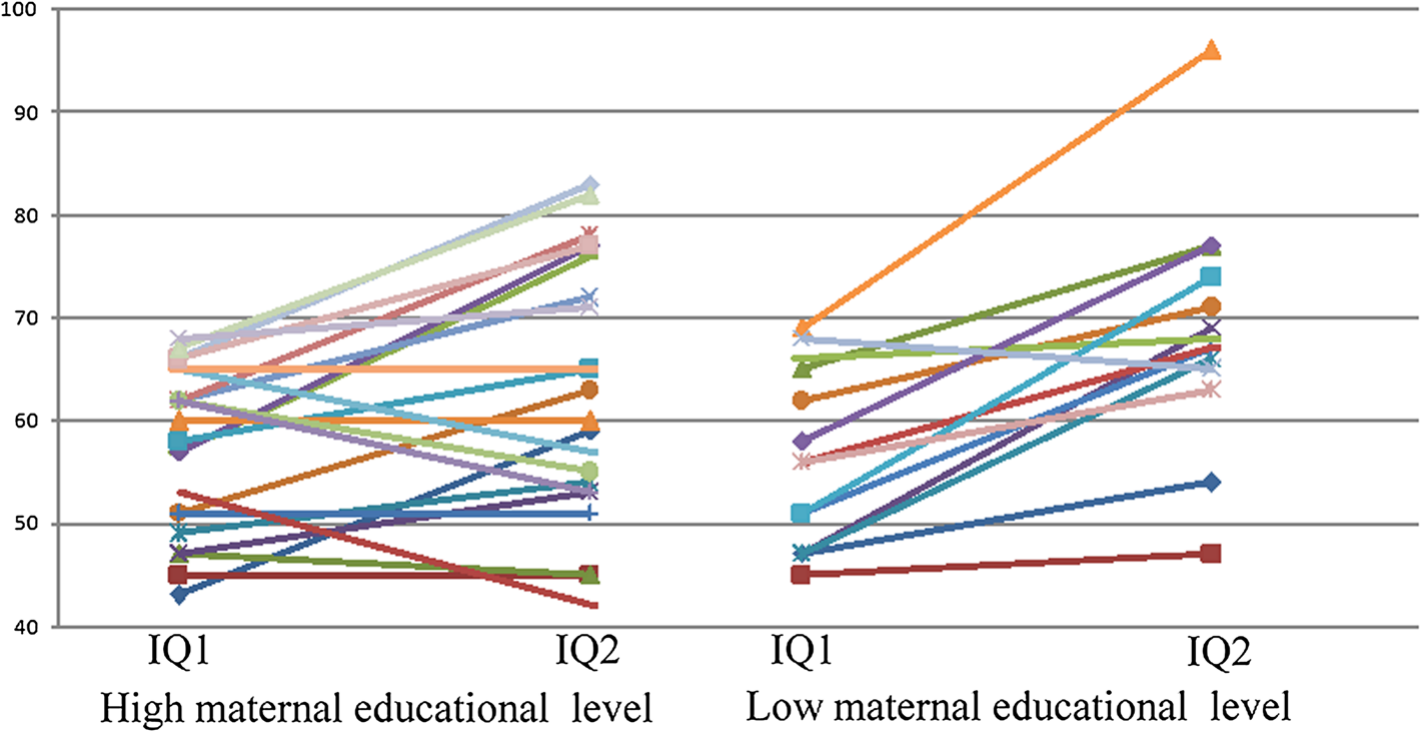
Changes in IQ (IQ2-IQ1) by maternal education level.

**Figure 2:**
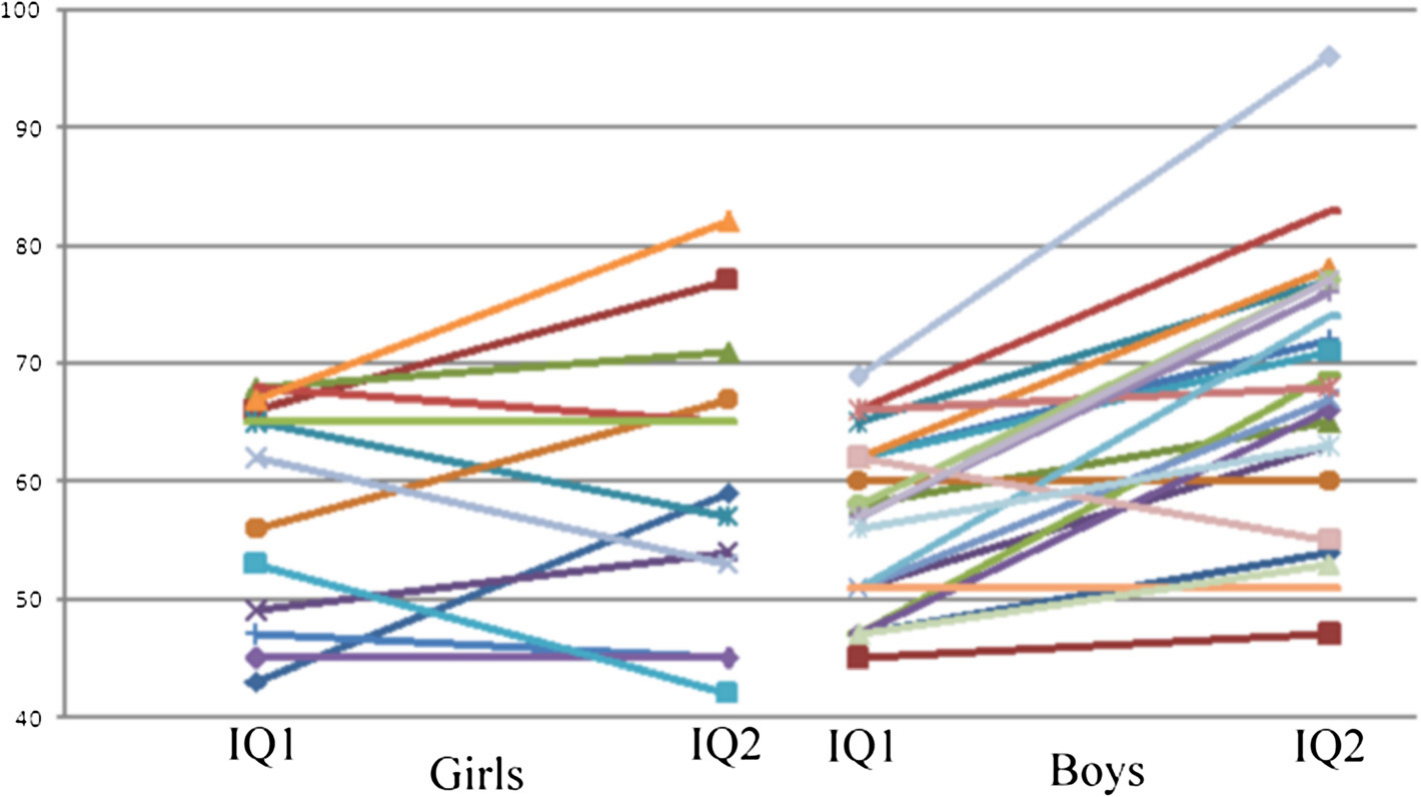
Changes in IQ (IQ2-IQ1) by gender.

Except for participants from single-parent families, for which the difference reached only marginal statistical significance (*p* = 0.104), IQ2 was significantly higher than IQ1 in all participants when they were divided into groups according to environmental risk factors, no matter they had the risk factor or not (Table 1). For all the five environmental risk factors, we observed a higher IQ2–IQ1 in the positive group. In particular, the difference was significant for “low maternal educational level” (12.4 *vs.* 5.5, *p =* 0.047) (Figure 1). The difference was also significant for male gender (11.5 *vs.* 2.2, *p =* 0.005) (Figure 2).

**Table 1:** Associations between environmental risk factors and IQ scores

**Risk Factors**	**IQ1**	**IQ2**	**IQ2-IQ1**	***p***^**a**^
	**Mean (SD)**	**Mean (SD)**	**Mean (SD)**	
Gender				
Boy (n = 23)	56.4 (7.3)	67.9 (11.5)	11.5 (8.7)	<0.001*
Girl (n = 13)	58.0 (9.5)	60.2 (12.5)	2.2 (9.0)	0.406
*p*^b^	0.515	0.081	0.005*	
Test interval (median = 16 month)				
≤ Median (n = 19)	57.6 (6.3)	65.5 (10.6)	7.9 (9.3)	0.005*
> Median (n = 17)	56.2 (9.8)	64.6 (14.3)	8.4 (10.7)	0.002*
*p*^b^	0.827	0.827	0.975	
Low maternal education level				
Yes (n = 14)	56.3 (8.5)	68.6 (11.3)	12.4 (9.0)	<0.001*
No (n = 22)	57.4 (7.9)	62.9 (12.6)	5.5 (9.6)	0.014*
*p*^b^	0.708	0.211	0.047*	
Low father’s education level				
Yes (n = 10)	57.0 (8.5)	67.5 (13.9)	10.5 (9.8)	0.008*
No (n = 26)	57.0 (8.1)	64.2 (11.8)	7.2 (9.9)	0.001*
*p*^b^	0.887	0.621	0.367	
Low income				
Yes (n = 4)	55.0 (8.8)	66.8 (15.5)	11.8 (7.3)	0.048*
No (n = 32)	57.2 (8.1)	64.9 (12.1)	7.7 (10.1)	0.001*
*p*^b^	0.667	0.743	0.449	
Single-parent family				
Yes (n = 5)	48.8 (5.2)	58.2 (14.1)	9.4 (10.6)	0.104
No (n = 31)	58.3 (7.7)	66.2 (11.9)	7.9 (9.9)	<0.001*
*p*^b^	0.016*	0.234	0.819	
Grand parenting				
Yes (n = 6)	56.8 (9.7)	67.7 (18.2)	10.8 (9.5)	0.039*
No (n = 30)	57.0 (7.9)	64.6 (11.1)	7.6 (10.0)	<0.001*
*p*^b^	0.983	0.832	0.580	

^a^Wilcoxon signed ranks test or paired t test.

^b^Mann-Whitney U test.

*p <0.05.

Using univariate linear regression, we found “low maternal educational level” (β = 6.9, *p =* 0.038), male gender (β = 9.4, *p =* 0.004), and a younger age at the first test (β = -0.6, *p =* 0.045) were significant predictors of the effectiveness (IQ2–IQ1) (Table 2). In multi-variate regression analysis, “low maternal educational level”, male gender, and younger age at the first test were significant independent predictors of the effectiveness. After adjusting for age at the first test, a low maternal educational level was associated with a 7.1 increase in IQ2 (*p =* 0.026), and male gender was associated with a 10.4 increase in IQ2 (*p =* 0.001). Because there was a significant correlation between low maternal educational level and male gender (r = 0.36, p = 0.030), we did put them into a model at the same time but evaluated their effects separately. After adjusting for maternal educational level each month older at the first test was associated with a 0.6 decrease in IQ2 (*p =* 0.030), and after adjusting for gender each month older at the first test was associated with a 0.7 decrease in IQ2 (*p =* 0.006). Whereas there were positive associations between the effectiveness and the numbers of hours per weeks on treatments at the hospital and kindergarten education, the associations did not reach statistical significance.

**Table 2:** Regression analyses on effectiveness of early intervention

**Factor**	**Uni-variate Analysis**		**Multi-variate Analysis**^**a**^			
			**Model 1**		**Model 2**	
	***β***	***p***	**Adjusted *β***	***p***	**Adjusted *β***	***p***
Age at the first test (month)	−0.6	0.045*	−0.6	0.030*	−0.7	0.006*
Male gender	9.4	0.004*			10.4	0.001*
Hospital treatment (hour/week)	1.0	0.713				
Kindergarten education (hour/week)	0.1	0.546				
Environmental risk factors						
Low maternal education level	6.9	0.038*	7.1	0.026*		
Low father’s education level	3.3	0.379				
Low income	4.1	0.444				
Single-parent family	1.5	0.762				
Grand parenting	3.2	0.470				

^a^The model included variables with statistical significance only.

*p < 0.05.

## Discussion

Our study results support the effectiveness of early intervention on children with ID, and the increase of 8.1 in IQ was compatible with reports from other countries [17,28]. Students with an IQ less than 70 generally need special education at primary and secondary schools. In our study, 13 of the 36 participants (36.1%) with an IQ less than 70 had an improved IQ that was higher than 70 in the second test, which means that early intervention can reduce the need for special education and thus save costs. In the U.S., for example, special education costs the government 6000 dollars each year on each student [29]. Therefore, even though early intervention costs time and money, it is worthy when the effort and costs spent on special education later are taken into consideration [16].

While previous studies were inconclusive on whether early intervention in an earlier age is more effective [11,13], the current study found a younger age at the first test was associated with a larger improvement. Although some cases in our study might have received certain treatment and kindergarten education before the first WPPSI-R test, without the services provided by the social workers, complete clinical evaluation, and comprehensive developmental or intellectual tests, such treatment and education can hardly be regarded as formal early intervention. Therefore, it is reasonable to adopt the time of the first test as the beginning of early intervention [24].

Among the environmental risk factors we evaluated, low maternal educational level was the most prevalent. The larger improvement in participants with low maternal educational levels we observed is compatible with findings from previous studies [14,19,21]. This reminds us that children with a low maternal educational level should be placed at higher priorities for early intervention [19,20]. Of the seven mothers with foreign nationalities in our study, six had low educational levels, which constituted 42.9% of all cases with a low maternal educational level. This has become a concern to the educators in Taiwan [30]. In our study, early intervention was more effective in boys than in girls, and this is compatible with findings in the Chicago Child–parent Center Program on 5-year-old children [31,32].

Whereas the positive association between the number of hours of intervention and the effectiveness of early intervention in our study did not reach statistical significance, many studies in Western countries observed such an association [11,15]. We believe the quality of early intervention in the Chia-Yi area may not be as good as those in Western countries, making the effects smaller.

The early intervention program in Taiwan generally includes (1) reporting, referral, and management of cases, mainly by the social workers, (2) clinical evaluation, mainly by the physicians, and (3) treatment and education, mainly by physical therapists, occupation therapists, speech therapists, clinical psychologists, and kindergarten teachers. These professionals work in three different fields: social welfare, healthcare, and education, and the cooperation among them has been a key issue in early intervention. The current study is a result of such cooperation.

We recognized several limitations of our study. Due to ethical considerations, it was not feasible to have a control group who were not given early intervention [13]. Furthermore, because early intervention is very popular in Taiwan, it is very difficult to recruit a control group consisting of children who did not receive early intervention [28]. In fact, even if we were able to gather a substantial number of such controls, factors affecting the receipt of early intervention are very likely to affect the effectiveness of the intervention and thus become confounders in the study. Whereas we have included all the qualified candidates in the area, the number of the participants was not large, and further studies in other areas are needed to confirm the generalizability of our study results. In addition, the data were collected retrospectively, and therefore we relied on recorded information. Furthermore, using WPPSI-R as the tool for evaluation, we recruited cases who received early intervention at relatively older ages. Moreover, we evaluate only the changes in intellectual functioning but not the changes in adaptive functioning.

## Conclusions

This study showed that early intervention can improve the IQ of children with ID, and the earlier the intervention the better. In addition, the effectiveness is more prominent in boys and in children with a low maternal educational level. Nonetheless, we evaluate the effectiveness at relatively short periods of time, and therefore further follow-ups are necessary to evaluate the long-term effects and effects on verbal and performance separately. In addition, studies on patients of younger ages and studies with larger numbers of boys and girls are needed to confirm our findings.

### Consent

The parents or legal guardians of all the participants have given their consents for the participation of their children in this study.

## Competing interests

The authors declare that they have no competing interests.

## Authors’ contributions

DCL and CHC conceived the study, and YCT and HRG help design the study. DCL, YMH, JHL, and SFY recruited the patients and performed the evaluation. DCL and HRG analyzed the data, and YCT help draft the manuscript. All authors read and approved the final manuscript.

## Pre-publication history

The pre-publication history for this paper can be accessed here:

http://www.biomedcentral.com/1471-2431/14/170/prepub
